# Homozygous CREM-IbΔC-X Overexpressing Mice Are a Reliable and Effective Disease Model for Atrial Fibrillation

**DOI:** 10.3389/fphar.2018.00706

**Published:** 2018-07-05

**Authors:** Frank T. Stümpel, Juliane Stein, Kirsten Himmler, Beatrix Scholz, Matthias D. Seidl, Boris V. Skryabin, Frank U. Müller

**Affiliations:** ^1^Institut für Pharmakologie und Toxikologie, Westfälische Wilhelms-Universität Münster, Münster, Germany; ^2^Core Facility TRAnsgenic Animal and Genetic Engineering Models (TRAM), University of Münster, Münster, Germany

**Keywords:** atrial fibrillation, atrial hypertrophy, animal models of disease, antiarrhythmics, cAMP response element modulator

## Abstract

**Background:** Atrial fibrillation (AF) is a significant cause of morbidity and mortality with foreseeably increasing prevalence. While large animal models of the disease are well established but resource intensive, transgenic AF mouse models are not yet widely used to develop or validate novel therapeutics for AF. Hemizygous mice with a cardiomyocyte-specific overexpression of the human cAMP response element modulator (CREM) isoform IbΔC-X spontaneously develop AF on grounds of an arrhythmogenic substrate consisting of alterations in structure, conduction, and calcium handling.

**Objective:** We investigated if homozygous expression of the CREM-IbΔC-X transgene in mice alters the time course of AF development, and if homozygous CREM-IbΔC-X transgenics could be suitable as a disease model of AF.

**Methods:** Southern Blot, quantitative real-time PCR, and immunoblotting were used to identify and verify homozygous transgenics. Cardiac gravimetry, quantitative real-time RT-PCR, histology, survival analysis, and repeated ECG recordings allowed assessment of phenotypic development and effects of antiarrhythmic drugs.

**Results:** Homozygous animals could be identified by Southern blot and quantitative PCR, showing a strong trend to increased transgenic protein expression. In homozygous animals, atrial hypertrophy appeared earlier and more pronounced than in hemizygous animals, going along with an earlier onset of spontaneous AF, while no increased early mortality was observed. Application of a rate-controlling drug (esmolol) led to the expected result of a decreased heart rate. Application of a rhythm-controlling drug (flecainide) showed effects on heart rate variability, but did not lead to a definitive conversion to sinus rhythm.

**Conclusion:** We suggest homozygous CREM-IbΔC-X overexpressing mice as a reliable model of early onset, rapidly progressive AF.

## Introduction

In most developed countries, atrial fibrillation (AF) is a significant cause of morbidity and mortality, often due to its thromboembolic complications (Wolf et al., 1998). Because the prevalence of this disease rises with age its importance as therapeutic challenge is expected to increase further (Chugh et al., 2014). Presently, pharmacological antiarrhythmic treatment options are limited to drugs with significant adverse effects and contraindications. If catheter ablation or surgical options are unavailable, not feasible, or unsuccessful, most patients will ultimately remain in permanent AF and need lifelong antithrombotic therapy, which itself carries risks and remains a balancing act between embolism and bleeding (Gallagher et al., 2014). To gain insights into the mechanisms of AF pathophysiology, as well as to develop and test new antiarrhythmic substances to treat AF, reliable animal models are needed. To date, most AF models used are large animal models (dogs, goats, and pigs) which had their AF caused by intervention (tachypacing, artificial mitral insufficiency, papillary muscle ischemia, etc.) (Nishida et al., 2010). Thus, these models need considerable resources in animal preparation and care.

More recently, several genetic mouse models exhibiting spontaneous AF have been described (Riley et al., 2012). However, many of these models show rapidly progressive phenotypes with ventricular involvement, leading to increased early mortality, while others develop AF only late in the process or with incomplete penetrance.

CREM-IbΔC-X belongs to the CREB/CREM/ATF group of cAMP-dependent transcription factors, which serve important functions in connecting cAMP-dependent signaling pathways to transcriptional activity of the cell (Müller et al., 2000; Kehat et al., 2006a; Altarejos and Montminy, 2011). CREM-IbΔC-X itself is a repressor isoform, initially identified in failing human hearts (Müller et al., 1998). Mice with a heart-directed overexpression of CREM-IbΔC-X revealed a striking phenotype with progressive atrial dilation and development of spontaneous AF (Müller et al., 2005). This phenotype could, at least in part, be attributed to the formation of an arrhythmogenic substrate, consisting of structural alterations, impaired conduction, and increased Ca^2+^ leak from the sarcoplasmic reticulum (Chelu et al., 2009; Kirchhof et al., 2013; Li et al., 2014; Seidl et al., 2017; Bukowska et al., 2018).

In this study we aimed to identify a possible relationship between the gene dose of CREM-IbΔC-X expression in the heart and the strength of the developed cardiac phenotype, as well as to evaluate a breeding line homozygous for CREM-IbΔC-X as a possible disease model of AF, e.g., for testing antiarrhythmic drugs.

## Materials and Methods

### Experimental Animals

Hemizygous animals transgenic for the HA-tagged CREM-IbΔC-X cDNA under control of the αMHC promoter were created as previously described. All animals used here were offspring of founder line Tg1 (Müller et al., 2005). To obtain animals homozygous for the transgene, two hemizygous transgenic animals were mated, and homozygous transgenics were identified by Southern Blot or quantitative real-time PCR. A breeding colony of homozygous transgenics was then established. Homozygous transgenics (CREM^T/T^) were compared with age-matched hemizygous (CREM^T/W^) and wild-type (WT) animals. All experiments were performed conforming to EU and national animal protection laws and regulations and as approved by the responsible authorities of the state of North Rhine-Westphalia.

### Southern Blot Analysis

Approximately 5 μg of genomic DNA purified from (tail) tissue samples was digested with restriction endonuclease EcoRV (Thermo Scientific Fermentas, Waltham, MA, United States), fractionated on 0.8% agarose gels, and transferred to GeneScreen nylon membranes (PerkinElmer, Waltham, MA, United States). The membranes were hybridized with a ^32^P-labeled 2.2 kb probe containing sequences PCR-amplified from the CREM-IbΔC-X transgene, using primers 5′-TAGCCCACACCAGAAATGACAGAC-3′ (forward) and 5′-GTGGTGTGACATAATTGGACAAACTAC-3′ (reverse), and washed with (final concentrations) 0.5x SSPE (1x SSPE is 0.18 M NaCl, 10 mM NaH_2_PO_4_, and 1 mM EDTA [pH 7.7]) and 0.5% (w/v) sodium dodecyl sulfate at 65°C.

### DNA Quantification by Quantitative Real-Time PCR

Purified genomic DNA was used as template. Real-time qPCR was performed in a LightCycler carousel-type device (Roche, Mannheim, Germany) using the LightCycler FastStart DNA Master SYBR Green I kit (Roche). Primers used to identify CREM-BHIb-cDNA were (forward) 5′-CCAAAATTCAATCCCTGCTT-3′ and (reverse) 5′-TCGACATTCTTTGGCAGCTT-3′. Primers were exon-spanning to exclude the amplification of wild-type CREM DNA containing introns. Quantification was performed using LightCycler software v. 3.5 (Roche), and statistical analysis was done using REST Software v. 2009 (M. Pfaffl, TU Munich, Germany and Qiagen, Hilden, Germany).

### mRNA Quantification by Quantitative Real-Time RT-PCR

Total quantification of mRNA levels was performed as previously described (Seidl et al., 2017) with following modifications: Briefly, RNA was extracted from both atria using TRIzol^®^ (Thermo Fisher) and Direct-zol^TM^ RNA MiniPrep (Zymo Research, Irvine, CA, United States) according to the manufacturer’s instructions. Samples of 0.9 μg RNA were randomly reversely transcribed to cDNA using Transcriptor First Strand cDNA synthesis kit (Roche) and quantitative real time RT-PCR was carried out using a LightCycler 480 System (Roche). Relative quantification was performed by calculating relative expression ratios using the ΔΔ*Ct* method and the relative expression software tool (REST Software v. 2009). Hypoxanthine-guanine phosphoribosyltransferase (*Hprt*) was used as a reference gene. Primers used were: *Akap9* (A-kinase anchor protein 9) fwd 5′-AAGGAATGCGAGACCCTGAA-3′, rev 5′-GACAGCCTTCACTAACCCCT-3′, *Ank2* (ankyrin 2, brain) fwd 5′-AGGTGGTCAGATTGATGCCA-3′, rev 5′-GCCTTGTATTGGAGCAGGTG-3′, *Cacna1c* (calcium channel, voltage-dependent, L type, alpha 1C subunit [Ca_v_1.2]) fwd 5′-GCTCTCTTCACCGTCTCCAC-3′, rev 5′-GACGAAACCCACGAAGATGT-3′, *Cav3* (caveolin 3) fwd 5′-CAGCCTCACAATGATGACCG-3′, rev 5′-TTCCATACACCGTCGAAGCT-3′, *Cxadr* (coxsackie virus and adenovirus receptor) fwd 5′-ATGGCTGATATCCCCGTCTG-3′, rev 5′-CTCTTCCGATCCATCCACGA-3′, *Fxyd1* (FXYD domain-containing ion transport regulator 1) fwd 5′-GAACCGGATCCATTCACCTA-3′, rev 5′-TTCCCCAGTTCTCTGCTGTT-3′, *Gja5* (gap junction protein, alpha 5 [connexin 40]) fwd 5′-AGAGCCTGAAGAAGCCAACT-3′, rev 5′-GGCGTGGACACAAAGATGA-3′, *Kcnd2* (potassium voltage-gated channel, Shal-related family, member 2 [K_v_4.2]) fwd 5′-CTGCTCACGGAGACACAAAA-3′, rev 5′-CGGCTGTTGGATAGTGGAGT-3′,*Kcne1* (potassium voltage-gated channel, Isk-related subfamily, member 1) fwd 5′-TTCTTCACCCTGGGCATCAT-3′, rev 5′-CCGCCTGGTTTTCAATGACA-3′, *Kcnv2* (potassium channel, subfamily V, member 2 [K_v_11.1]) fwd 5′-TCTCTGCAGCTGTCTACTCG-3′, rev 5′-AATAATGCCAAAAGCGATGC-3′, *Mef2c* (myocyte enhancer factor 2C) fwd 5′-ACCAGGACAAGGAATGGGAG-3′, rev 5′-GGCGGCATGTTATGTAGGTG-3′, *Myl3* (myosin, light polypeptide 3) fwd 5′-CCGAGTTTGATGCCTCCAAG-3′, rev 5′-CTTCCTGTTTTGGCTTCCCC-3′, *Scn5a* (sodium channel, voltage-gated, type V, alpha [Na_v_1.5]) fwd 5′-TACCGCATAGTGGAGCACAG-3′, rev 5′-ATCTCGGCAAAGCCTAAGGT-3′.

### SDS-PAGE and Quantitative Immunoblotting

Heart tissue was homogenized in a buffer containing NaHCO_3_ and SDS as previously described (Rockman et al., 1996). Proteins were electrophoresed under reducing conditions in SDS-PA gels in Hoefer SE 600 chambers and blotted to nitrocellulose membranes (GE Healthcare, Little Chalfont, United Kingdom). HA-tagged CREM isoforms were detected using an anti-HA-antibody (1:1000; Cell Signaling, Danvers, MA, United States), an HRP-conjugated anti-rabbit-IgG 2nd antibody (1:5000; GE Healthcare), and the enhanced chemiluminescence (ECL) system (Promega, Madison, WI, United States). Images were acquired and quantitatively analyzed using a ChemiDoc MP system and Image Lab software (Bio-Rad, Hercules, CA, United States).

### Histology and Quantification of Fibrosis

Masson’s trichrome staining was performed on 5 μm sections of paraffin embedded hearts for estimation of interstitial fibrosis as previously described (Bukowska et al., 2018). Whole heart sections were imaged by light microscopy using NIS-Elements software (Nikon, Tokyo, Japan) and fibrotic areas within the ventricle (excluding the cardiac skeleton and the valves) were detected and quantified using Image-Pro Plus software (Media Cybernetics Inc., Rockville, MD, United States).

### Electrocardiography

Mice were anesthetized with isoflurane (∼1.2% v/v) and nitrous oxide (∼66% v/v) while being placed on a warmed pad in supine position. Needle electrodes were attached to obtain limb leads (Einthoven) and augmented limb leads (Goldberger). ECGs were recorded and analyzed electronically using PowerLab hardware and LabChart Pro software (ADInstruments, Bella Vista, Australia). Where required by the experimental protocol, a tube catheter was inserted into the left external jugular vein and drugs, i.e., the β_1_-adrenoceptor antagonist (class II antiarrhythmic) esmolol (Baxter, Unterschleißheim, Germany) 10 mg/kg bw or the Na^+^-channel blocker (class Ic antiarrhythmic) flecainide (MEDA Pharma, Bad Homburg, Germany) 5 mg/kg bw, were administered intravenously using an automated syringe pump (B. Braun, Melsungen, Germany). After invasive procedures, animals were euthanized by CO_2_ inhalation without having regained consciousness. Hearts were excised, photographed, weighed, and stored at -80°C until further analysis.

### Analysis of Onset of Atrial Fibrillation and Overall Survival

Animals were monitored weekly by ECG for the occurrence of atrial fibrillation. The first occurrence of AF was noted as event. Animals that could not be followed further were censored. For survival analysis, only deaths from natural causes were counted as events. Survival without AF and overall survival time were estimated using the Kaplan–Meier estimator and compared using the log-rank test.

### Statistical Analysis

Data are given as mean ± standard error of the mean (SEM), geometric mean and 95% confidence interval, or median and interquartile range, depending on the type of data, as specified in the “Results” section. “*n*” indicates the number of independent experiments. Two groups of normally distributed data were compared by student’s *t*-test. More than two groups of normally distributed data were compared by one-way ANOVA, with *post hoc* testing done by the Holm–Sidak method. Two groups of data from repeated measurements were compared using the paired *t*-test. More than two groups of data from repeated measurements were compared by one-way RM ANOVA. Groups of non-normally distributed data were compared by Kruskal–Wallis ANOVA on Ranks, with *post hoc* testing done by Dunn’s method. Groups of time-to-event data (like survival) were compared using the Kaplan–Meier estimator and the log-rank test, with *post hoc* testing done by the Holm–Sidak method. Gene expression data were compared using the ΔΔ*Ct* randomization method. The SigmaStat package (Systat Software Inc., San Jose, CA, United States) was used to perform all statistical analyses, except for the ΔΔ*Ct* randomization method, which was done using REST software (see above).

## Results

### Definite Identification of Homozygous Transgenic Animals by Southern Blot or Quantitative Real-Time PCR

**FIGURE 1:**
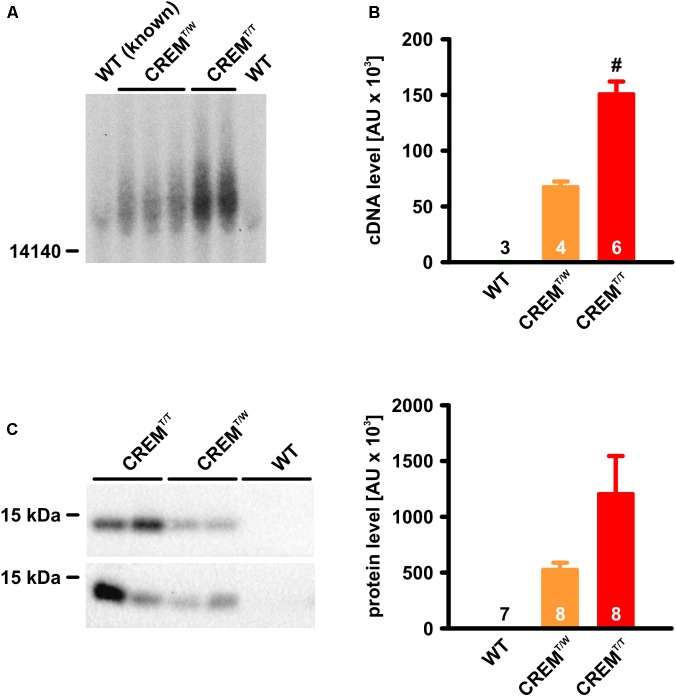
Identification and verification of CREM^T/T^ animals. **(A)** Southern Blot. Note that two of the positive samples yielded much stronger signals than the others. These samples were identified as CREM^T/T^. **(B)** Quantitative real-time PCR from genomic DNA. Note that, while the transgenic sequence was virtually undetectable in WT, its detection in CREM^T/T^ was about twice as high as in CREM^T/W^. (^#^*p* < 0.05 vs. CREM^T/W^). **(C)** Detection of HA-tagged CREM-IbΔC-X protein expression by western blot. Note that CREM^T/T^ hearts expressed the transgenic product about twice as highly as CREM^T/W^ hearts (CREM^T/T^ vs. CREM^T/W^ *p* = 0.09). All quantified data are given as mean ± SEM.

To identify possible homozygous transgenic animals among the offspring of two hemizygous transgenic parents, genomic tail-tip DNA was digested with the restriction endonuclease EcoRV and hybridized to a 2.2 kb probe containing the ORF of the human CREM-IbΔC-X cDNA, obtained by PCR from the transgenic construct (**Figure 1**). As the introduced transgene (7868 b) does not contain EcoRV restriction sites, hybridization to DNA fragments larger than ∼8 kb was expected. Indeed, samples of known transgenic animals yielded signals of more than 15 kb size, probably due to multiple consecutive insertions of the transgene. Two of the transgenic animal samples showed much stronger signals than the others, identifying these animals as homozygous transgenics.

(In wild-type animals a much weaker signal was visible, probably due to hybridization of the probe to mouse CREM exons H or Ib [loci chr18:3273422-3273581 and chr18:3267589-3267732, GRCm38/mm10 mouse genome assembly], which are more than 90% homologous with the human sequence. In EcoRV-digested genomic mouse DNA, these loci are expected to be part of a 35828 b fragment).

As a second means of verification of CREM^T/T^ animals, quantitative real-time PCR was employed, using genomic DNA as template. Primers were binding over exon boundaries within the transgene sequence, thus being specific to CREM-IbΔC-X cDNA lacking introns. Calculated transgene DNA content in CREM^T/T^ samples (150600 ± 11365 arbitrary units, AU, *n* = 6) was about twice as high as in CREM^T/W^ samples (67280 ± 5061 AU, *p* < 0.05, *n* = 4, Student’s *t*-test). In WT samples, the transgene sequence was virtually undetectable (3 ± 1 AU, *n* = 3).

As a further control of homozygosity, homozygous transgenic animals identified by one of these means were mated with wild-types, which, without exception, only led to transgenic progeny in 13 litters.

### Increased CREM-IbΔC-X Protein Expression in Homozygous Transgenic Animals

To investigate if the homozygous existence of the transgene goes along with an increased expression of its protein products, ventricle homogenates were electrophoresed in SDS-PA-gels and blotted to nitrocellulose membranes, and CREM proteins being translated from the transgene were immunologically detected by their HA-tags (**Figure 1**). As expected from earlier experiments, a 14 kDa-protein was detected in transgenic samples, putatively corresponding to HIbII (Müller et al., 2005). While no expression was detected in WT (*n* = 7), expression of this protein in CREM^T/T^ (1203191 ± 340900 AU, *n* = 8) was about twice as high as in CREM^T/W^ (524054 ± 64820 AU, *n* = 8, *p* = 0.09, Student’s *t*-test).

### Earlier and More Pronounced Atrial Hypertrophy as Well as Ventricular Hypertrophy in Homozygous Transgenic Animals

**FIGURE 2:**
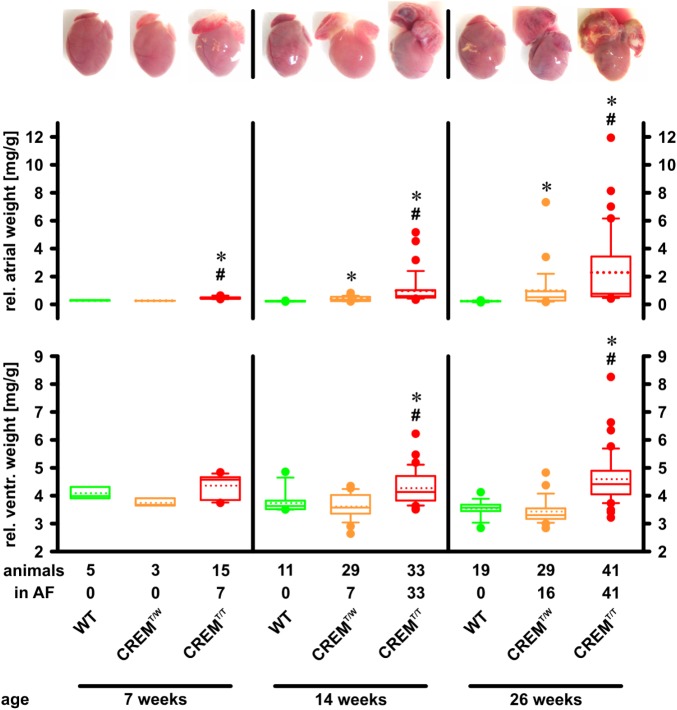
Development of atrial and ventricular hypertrophy. Hearts were excised, rinsed, and photographed, and atria and ventricles were weighed. Atrial and ventricular weights are given relative to body weight. Note that atrial hypertrophy developed earlier and was more profound in CREM^T/T^ than in CREM^T/W^. Further note that in addition to the earlier onset of atrial hypertrophy in CREM^T/T^ at 7 weeks of age, AF had also appeared already at this age, while in CREM^T/W^ it was not yet detected. CREM^T/T^ also developed ventricular hypertrophy, whereas CREM^T/W^ did not. ^∗^*p* < 0.05 vs. WT; ^#^*p* < 0.05 vs. CREM^T/W^. Boxes represent median and interquartile range; whiskers show the 10th and 90th percentiles; the dotted line is the mean.

Overexpression of CREM-IbΔC-X protein products leads to marked atrial hypertrophy (Müller et al., 2005). To assess if the increased transgene expression in homozygous transgenics alters time course and level of hypertrophy, hearts of WT, CREM^T/W^, and CREM^T/T^ animals were photographed and weighed at three different ages (7, 14, and 26 weeks), and atrial and ventricular weights relative to body weight were calculated (**Figure 2**). At 7 weeks of age already, relative atrial weights of CREM^T/T^ (0.44 [0.40–0.50] mg/g, median and interquartile range, *n* = 15) were significantly (*p* < 0.05, ANOVA on Ranks) increased when compared to CREM^T/W^ (0.25 [0.25–0.26] mg/g, *n* = 3) or WT (0.27 [0.26–0.31] mg/g, *n* = 5), while the latter two groups were not different from each other. At 14 and 26 weeks of age, significant (*p* < 0.05 vs. WT) atrial hypertrophy had developed also in CREM^T/W^, but CREM^T/T^ showed much more distinct atrial hypertrophy, being significantly increased as compared to both former groups (14 weeks: WT 0.22 [0.21–0.25] mg/g, *n* = 11, CREM^T/W^ 0.34 [0.24–0.54] mg/g, *n* = 29, CREM^T/T^ 0.6 [0.48–1.02] mg/g, *n* = 33; 26 weeks: WT 0.24 [0.22–0.25] mg/g, *n* = 19, CREM^T/W^ 0.51 [0.27–0.93] mg/g, *n* = 29, CREM^T/T^ 0.77 [0.57–3.43] mg/g, *n* = 41). In addition to atrial hypertrophy, CREM^T/T^ also showed significant ventricular hypertrophy, being detectable at 14 and 26 weeks of age, while no significant differences were detected between CREM^T/W^ and WT (7 weeks: WT 3.99 [3.91–4.32] mg/g, *n* = 5, CREM^T/W^ 3.67 [3.65–3.91] mg/g, *n* = 3, CREM^T/T^ 4.58 [3.84–4.66] mg/g, *n* = 15; 14 weeks: WT 3.63 [3.52–3.82] mg/g, *n* = 11, CREM^T/W^ 3.57 [3.36–4.03] mg/g, *n* = 29, CREM^T/T^ 4.13 [3.83–4.71] mg/g, *n* = 33; 26 weeks: WT 3.57 [3.45–3.67] mg/g, *n* = 19, CREM^T/W^ 3.3 [3.17–3.54] mg/g, *n* = 29, CREM^T/T^ 4.42 [4.05–4.89] mg/g, *n* = 41). Body weights were unchanged (1-way ANOVA) between groups at all three ages (7 weeks: WT 27.26 ± 2.22 g, mean ± SEM, *n* = 5, CREM^T/W^ 24.47 ± 2.28 g, *n* = 3, CREM^T/T^ 22.91 ± 0.96 g, *n* = 15; 14 weeks: WT 30.31 ± 1.26 g, *n* = 11, CREM^T/W^ 29.59 ± 0.87 g, *n* = 29, CREM^T/T^ 29.88 ± 0.86 g, *n* = 33; 26 weeks: WT 38.2 ± 1.07 g, *n* = 19, CREM^T/W^ 35.97 ± 1.09 g, *n* = 29, CREM^T/T^ 35.17 ± 0.92 g, *n* = 41). Summing up, in CREM^T/T^ atrial hypertrophy appeared earlier and much more pronounced than in CREM^T/W^. Ventricular hypertrophy was also detected in CREM^T/T^, while being absent in CREM^T/W^ and WT.

### Earlier Onset of Atrial Fibrillation in Homozygous Transgenic Animals

**FIGURE 3:**
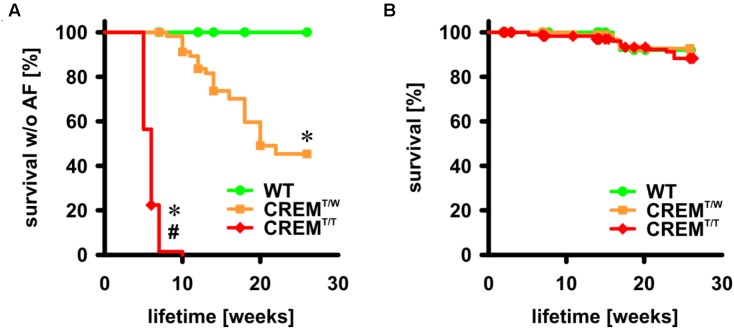
Onset of AF and overall survival up to the age of 26 weeks. **(A)** Animals were followed by once-weekly ECG and the first appearance of spontaneous AF was recorded as event. Animals not available for further examination were censored. Note that all CREM^T/T^ had developed AF by the age of 10 weeks, more than 50% of CREM^T/W^ had developed AF by the age of 26 weeks, and no WT developed AF up to the age of 26 weeks. WT: *n* = 35, 100% censored; CREM^T/W^: *n* = 65, 66% censored; CREM^T/T^: *n* = 85, 4% censored ^∗^*p* < 0.05 vs. WT, ^#^*p* < 0.05 vs. CREM^T/W^. **(B)** Deaths in the colony from natural causes were recorded as events. Note that there was no difference in survival between groups up to the age of 26 weeks. WT: *n* = 52, 96% censored, CREM^T/W^: *n* = 100, 95% censored, CREM^T/T^: *n* = 197, 93% censored.

Mice overexpressing CREM-IbΔC-X develop spontaneous atrial fibrillation (Müller et al., 2005). A cohort of animals (the same as used to assess atrial and ventricular hypertrophy, with a total of 85 CREM^T/T^, 65 CREM^T/W^, and 35 WT) were followed by weekly ECG to identify the onset of AF. We also noted spontaneous deaths in the colony (with a total of 197 CREM^T/T^, 100 CREM^T/W^, and 52 WT included into analysis) to estimate overall survival (**Figure 3**). In CREM^T/W^, more than 50% had developed AF by the age of 22 weeks (*p* < 0.05 vs. WT, log-rank test). In CREM^T/T^, more than 50% had developed AF already by the age of 6 weeks, and all animals had developed AF by the age of 10 weeks (*p* < 0.05 vs. CREM^T/W^ and WT). No appearances of AF were noted in WT. Overall survival of all three groups was comparable.

### Altered Gene Expression in Atria From Homozygous Transgenic Animals

**FIGURE 4:**
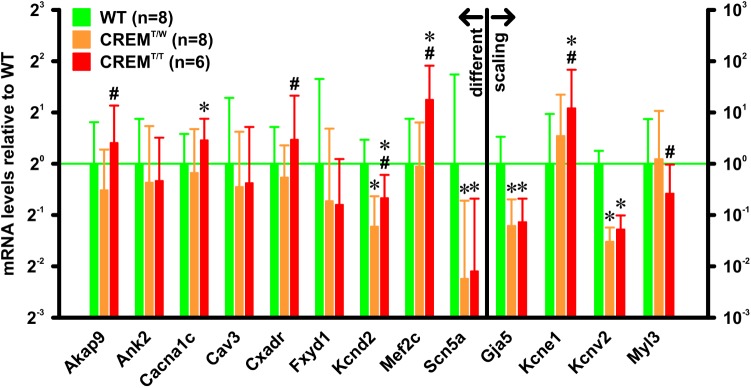
mRNA expression levels of selected genes. RNA was extracted from atria from 14 weeks old animals. Gene expression was quantified by quantitative real-time RT-PCR relative to Hprt as a reference gene. All expression levels are given relative to WT. Note that several genes were differently expressed in CREM^T/T^ vs. CREM^T/W^. ^∗^*p* < 0.05 vs. WT; ^#^*p* < 0.05 vs. CREM^T/W^. Data given as (geometric) mean and 95% confidence interval.

In atria of hemizygous transgenics, going along with the transition to hypertrophy and AF, a complex pattern and time course of gene regulation changes was found (Seidl et al., 2017). To get a first notion of the effects of the increased transgene expression in homozygous transgenics on gene regulation, mRNA levels of selected genes linked to AF development and identified in the hemizygous model were measured. RNA was extracted from atria of 14 weeks old WT (*n* = 8), CREM^T/W^ (*n* = 8), and CREM^T/T^ animals (*n* = 6), and gene expression was quantified by quantitative real-time RT-PCR relative to the expression of *Hprt* as reference (**Figure 4**). Of many of the investigated genes, expression was similar in CREM^T/W^ and CREM^T/T^, in that there was either no significant difference from WT in both groups (*Ank2* WT 1 [0.55–1.84], expression relative to WT and 95% confidence interval, CREM^T/W^ 0.77 [0.35–1.66], CREM^T/T^ 0.79 [0.48–1.42], *Cav3* WT 1 [0.41–2.44], CREM^T/W^ 0.73 [0.26–1.54], CREM^T/T^ 0.77 [0.3–1.64], *Fxyd1* WT 1 [0.32–3.15], CREM^T/W^ 0.6 [0.21–1.61], CREM^T/T^ 0.57 [0.27–1.07]) or both CREM^T/W^ and CREM^T/T^ were significantly (*p* < 0.05, ΔΔ*Ct* randomization test) different from WT without differences between the former groups (*Gja5* WT 1 [0.3–3.36], CREM^T/W^ 0.06 [0.02–0.2], CREM^T/T^ 0.07 [0.03–0.21], *Kcnv2* WT 1 [0.56–1.79], CREM^T/W^ 0.03 [0.02–0.06], CREM^T/T^ 0.05 [0.03–0.1], *Scn5a* WT 1 [0.3–3.34], CREM^T/W^ 0.21 [0.08–0.61], CREM^T/T^ 0.23 [0.09–0.62]). However, several genes were found to be regulated differently between CREM^T/W^ and CREM^T/T^, insofar as only CREM^T/T^ (and not CREM^T/W^) was significantly different from WT (*Cacna1c* WT 1 [0.67–1.5], CREM^T/W^ 0.88 [0.51–1.6], CREM^T/T^ 1.37 [1.01–1.84]) or significant differences were found between CREM^T/W^ and CREM^T/T^ (*Akap9* WT 1 [0.57–1.76], CREM^T/W^ 0.7 [0.43–1.21], CREM^T/T^ 1.32 [0.81–2.19], *Cxadr* WT 1 [0.61–1.64], CREM^T/W^ 0.83 [0.52–1.28], CREM^T/T^ 1.38 [0.81–2.51], *Kcnd2* WT 1 [0.72–1.38], CREM^T/W^ 0.43 [0.29–0.64], CREM^T/T^ 0.63 [0.45–0.86], *Kcne1* WT 1 [0.11–9.37], CREM^T/W^ 3.45 [0.49–22.24], CREM^T/T^ 11.98 [1.92–68.11], *Mef2c* WT 1 [0.54–1.84], CREM^T/W^ 0.96 [0.53–1.75], CREM^T/T^ 2.37 [1.48–3.76], *Myl3* WT 1 [0.14–7.43], CREM^T/W^ 1.22 [0.21–10.74], CREM^T/T^ 0.26 [0.04–0.96]).

### No Significant Increase in Ventricular Fibrosis in Homozygous Transgenic Animals

**FIGURE 5:**
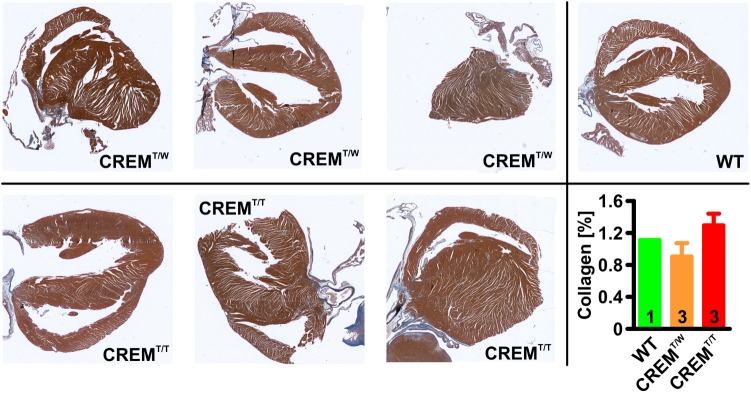
Quantification of ventricular fibrosis. 5 μm sections from hearts of 14 weeks old animals were stained with Masson’s trichrome, and interstitial and perivascular fibrosis in ventricles was measured by quantifying the ratio of the blue (i.e., collagen) areas relative to total areas of the ventricles (excl. the cardiac skeleton and the valves). Note that collagen content of both CREM^T/T^ and CREM^T/W^ was found to be comparable to that of one 14 weeks old WT animal, which was measured for reference, and not significantly different from each other. Data given as mean ± SEM.

To assess if homozygous transgenics develop an increased level of ventricular fibrosis, fibrosis (interstitial and perivascular) was measured in Masson’s trichrome stained histologic sections from hearts of 14 weeks old CREM^T/T^ and CREM^T/W^ (three animals per group) by quantifying the blue area (i.e., collagen) within the total ventricular area excl. the cardiac skeleton and the valves (**Figure 5**). Collagen content of both CREM^T/T^ and CREM^T/W^ was found to be comparable to that of one 14 weeks old WT animal, which was measured for reference, and not significantly different from each other (WT 1.11%, *n* = 1, CREM^T/W^ 0.9 ± 0.17%, mean ± SEM, *n* = 3, CREM^T/T^ 1.3 ± 0.15%, *n* = 3).

### Effects of Rate Controlling and Rhythm Controlling Drugs

**FIGURE 6:**
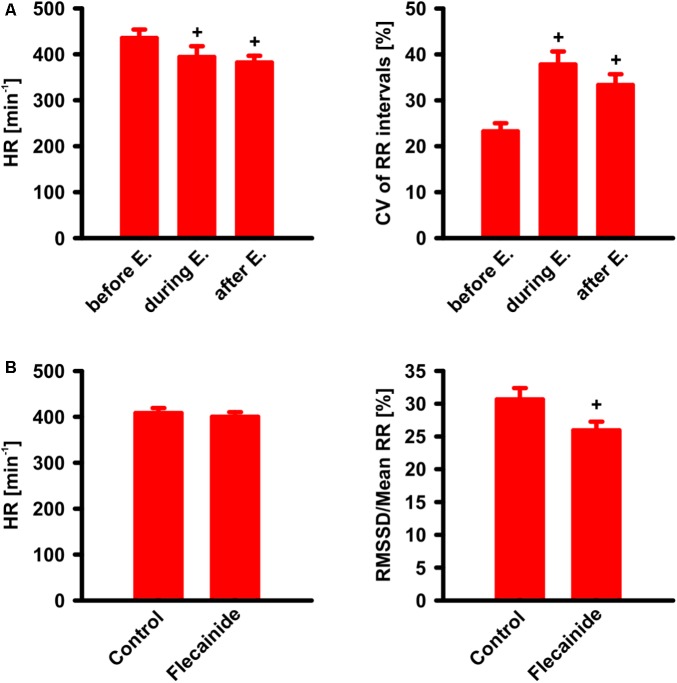
Drug effects on CREM^T/T^ animals with AF. **(A)** Effect of esmolol (E.) i.v. infusion (10 mg/kg/min during 1 min) on heart rate (HR) and coefficient of variation (CV) of RR intervals. Note that, as known and expected from humans, HR is found decreased during and shortly (2 min) after infusion, whereas CV of RR intervals was increased. ^+^*p* < 0.05 vs. before esmolol (*n* = 8). **(B)** Effect of flecainide. ECG was recorded during 90 min after i.v.-administration of flecainide (5 mg/kg) and compared to a previous control ECG (90 min) without flecainide administration. Note that beat-to-beat variability (measured as the root mean square of successive differences, relative to mean RR) was decreased after flecainide ^+^*p* < 0.05 vs. control (*n* = 15). All data given as mean ± SEM.

To examine the potential of the homozygous transgenic model for testing drugs commonly employed in the treatment of human AF, we treated 11 weeks old CREM^T/T^ animals with the β_1_-adrenoceptor antagonist esmolol (Vaughan Williams class II, 10 mg/kg) and 6–7 weeks old CREM^T/T^ animals with the Na^+^-channel blocker flecainide (Vaughan Williams class Ic, 5 mg/kg) while simultaneously recording ECGs (**Figure 6**). During and shortly (2 min) after esmolol infusion, heart rates were reduced (before infusion 435 ± 19 min^-1^ [mean ± SEM], during inf. 394 ± 24 min^-1^, after inf. 382 ± 17 min^-1^, *n* = 8, *p* < 0.05, RM ANOVA), while heart rate variability, measured as coefficient of variation of RR internals (CV, i.e., ratio of SD to mean) increased (before infusion 23 ± 2%, during inf. 38 ± 3%, after inf. 33 ± 2%, *p* < 0.05). Before, during, and after esmolol infusion, all examined animals were (and remained) in AF. To assess effects of flecainide, 90 min of ECG recordings immediately after flecainide administration were compared to (1–5 days) previously recorded control ECGs from the same animals. Over the course of 90 min, flecainide did not change heart rate (flecainide 400 ± 10 min^-1^, control 408 ± 11 min^-1^, *n* = 15). While no animal definitely converted to sinus rhythm during the 90-min follow-up, flecainide significantly reduced beat-to-beat RR variability, measured as the root mean square of successive differences, RMSSD, relative to mean RR (flecainide 26 ± 1%, control 31 ± 2%, *p* < 0.05, paired *t*-test). Furthermore, flecainide also tended to reduce RR interval randomness, measured by the turning point rate (Dash et al., 2009). The actual number of turning points tended to differ more from the number expected by randomness after flecainide treatment (16 ± 4 SDs) than without treatment (7 ± 2 SDs, *p* = 0.08, paired *t*-test).

## Discussion

### Relation Between Gene Dosage and Phenotype

In this study we describe the establishment of a breeding line homozygous for the insertion of human CREM-IbΔC-X cDNA. Creation of the original transgenic mouse model was described previously (Müller et al., 2005). During this original study, two independent founder lines were obtained, both of which showed the key phenotypic change of dilated atria going along with cardiac-specific expression of the CREM repressor isoform HIbII. As it is improbable that DNA insertion events occurred at the same loci in both founder lines it was thus reasonable to conclude that the atrial phenotype was indeed a consequence of transgene expression, and not due to unspecific gene insertion effects, such as disruption of other genes at insertion sites. However, one of the founder lines showed a markedly increased early mortality, which led to breeding failure. Whether this was caused by the many times stronger expression of CREM repressor isoforms or by other effects remained to be speculated. It was proposed (Davis et al., 2012) that transgenic models driven by the αMHC promoter should only be used with care to evaluate atrial phenotypes, as in the atria this promoter is already active throughout embryonic development, and massive overexpression of wild-type proteins alone could already lead to atrial hypertrophy, as was the case in a model overexpressing MYL3 (James et al., 1999). However, in this case, only one founder line grossly overexpressing the transgene showed this phenotype. Several other models overexpressing negative regulators of CRE-mediated transcription consistently showed atrial dilation (Fentzke et al., 1998; Okamoto et al., 2001; Kehat et al., 2006b), and downregulation of CREB target genes was connected to AF susceptibility in humans (Deshmukh et al., 2015). It therefore seems reasonable to suggest specific effects by which CREM-IbΔC-X overexpression contributes to the development of the atrial phenotype. We sought to generate animals homozygous for the insertion site of the transgene by mating hemizygous parents. Indeed, a Southern blot, which could also clearly identify wild-types, showed two different levels of transgenic DNA content in the genome of the progeny. As it is highly unlikely that more than one insertion site (or more specifically, more than one chromosome containing insertion sites) was left after several generations of breeding, this strongly hinted to a homozygous presence of this insertion site. This assumption could later be verified in offspring from breeding pairs only consisting of animals deemed homozygous, which showed doubled CREM-IbΔC-X cDNA content in a quantitative PCR when compared to transgenic offspring from the hemizygous breeding colony. Moreover, immunological detection of protein products translated from the transgene revealed that a product of the same size as in hearts from CREM^T/W^ animals could be identified in hearts from CREM^T/T^ animals, but in a roughly doubled quantity, albeit with greater variation between animals.

Going along with, and probably as a consequence of this increased expression of a CREM repressor isoform, the atrial phenotype developed more quickly and into a more pronounced state in CREM^T/T^ animals. While 7 weeks old CREM^T/W^ animals did not yet show signs of atrial hypertrophy, an increase in relative atrial weights was already apparent in CREM^T/T^ animals of the same age. At 14 and 26 weeks of age both hemizygous and homozygous animals had developed atrial hypertrophy, but also at this age hypertrophy was more distinct in CREM^T/T^, with the difference rather increasing with older age. Simultaneously, development of spontaneous atrial fibrillation was accelerated in CREM^T/T^. While slightly more than half of the hemizygous animals had shown episodes of spontaneous AF by the age of 22 weeks, all homozygous animals had developed spontaneous AF already by the age of 10 weeks. We performed a first comparative assessment of the levels of mRNAs encoding ion channels or gap junction proteins (*Cacna1c*, *Kcnd2*, *Scn5a*, *Kcne1*, *Kcnv2*, *Gja5*), ion channel regulators (*Fxyd1*), and proteins involved in ion channel localization and stabilization (*Ank2, Cav3*), structural and functional organization of intercalated disks (*Cxadr*), signal transduction (*Akap9)*, gene transcription (*Mef2c*), and myosin function (*Myl3*) to study the expression of genes linked to AF development and discussed in the context of the hemizygous model (Seidl et al., 2017). While many genes were regulated similarly in 14 weeks old CREM^T/W^ and CREM^T/T^ animals (vs. WT), some of the genes were only differently expressed in CREM^T/T^. Although the time course of gene expression changes and its interconnection with development of the AF phenotype was out of the scope of this study, it can be speculated that this was a consequence of the more advanced phenotype in the homozygous model. Thus, the homozygous model may well become a valuable addition to the hemizygous model also in future studies addressing molecular mechanisms of AF development.

In ventricular cardiomyocytes isolated from CREM^T/W^ hearts arrhythmogenic alterations in Ca^2+^-handling and K^+^-currents were identified, leading to an increased propensity to ventricular extrasystoles especially during β-adrenergic stimulation (Schulte et al., 2016). It is supposable that this might also be aggravated compared to CREM^T/W^. Nevertheless, overall survival of CREM^T/T^ and CREM^T/W^ animals was comparable up to the age of 26 weeks (and not different from WT), allowing the conclusion that there was no increased prevalence of life-threatening ventricular arrhythmias up to this age. In our present study, a mild ventricular hypertrophy (vs. WT) was found in CREM^T/T^ animals while being absent from CREM^T/W^. In the original study describing the hemizygous model (Müller et al., 2005), both newly established lines showed ventricular hypertrophy (Tg2 strongly, Tg1 mildly), going along with an increased ventricular function in Tg1 (Tg2 could not be measured due to early mortality and breeding failure, as mentioned above). In CREM^T/W^ (descending from Tg1), this hypertrophy probably regressed with decreasing gene dose due to breeding. Assessment of ventricular fibrosis in 14 weeks old CREM^T/T^ and CREM^T/W^ revealed no significant difference of interstitial and perivascular collagen content, both being well in the range reported for WT from the same background in another study (Seeland et al., 2007). In short, given these findings and the unchanged survival of CREM^T/T^ vs. CREM^T/W^, we have no reason to suspect overt ventricular pathology in CREM^T/T^, while we currently cannot formally rule it out.

In summary, this study indicates that in homozygous CREM-IbΔC-X transgenic animals a doubled gene dosage leads to an increased expression of gene products, which in turn accelerates and aggravates the atrial phenotype, yet without causing increased early mortality.

### Drug Effects

Drugs used as antiarrhythmic agents can be roughly categorized into those used for rhythm control and those used for rate control (Kirchhof et al., 2016). We evaluated one substance out of each group to verify that CREM^T/T^ animals adequately reproduce effects typically found in human AF. Esmolol is a short-acting β_1_-adrenoceptor antagonist, whose indications include quick achievement of rate control in AF patients with hemodynamically relevant tachycardia. Although our model, with a mean heart rate of about 400 min^-1^ under isoflurane anesthesia, is not as such mimicking this situation, a significant reduction of heart rate was observed during and shortly after esmolol infusion. Concurrently, heart rate variability was increased. Comparable results were obtained in a study whose authors treated human AF patients with a β-adrenoceptor antagonist: heart rates decreased, while heart rate variability increased, the latter effect being attributed to relative changes in vagal tone (van den Berg et al., 1997). Thus, to this extent, our model quite adequately reflects conditions in humans.

Flecainide is a Na^+^-channel blocker belonging to the class Ic antiarrhythmics, being effective in ventricular as well as supraventricular arrhythmias. In patients with non-permanent AF, but without concomitant structural ventricular disease, it can typically be used to attempt pharmacologic cardioversion. In this study, we applied flecainide to relatively young mice to ensure that the animals had not been in AF for a prolonged time. Nevertheless, in none of the experiments a definite conversion to sinus rhythm with visible P waves could be observed during 90 min of follow-up. Comparing ECGs recorded immediately after flecainide application to control ECGs recorded from the same animals few days previously, we found a decrease in beat-to-beat RR variability. At the same time, the dependence of RR interval duration on randomness was reduced by flecainide treatment. Both parameters, an increase in beat-to-beat RR variability as well as more randomly distributed RR interval durations, are used in an algorithm to detect the occurrence of AF in ECG recordings (Dash et al., 2009). The reduction of one of these parameters as well as the trend to reduction of the other might hint at a measurable atrial effect of flecainide in our AF model, yet not enough to induce conversion to sinus rhythm. However, flecainide was attributed vagolytic properties in a study on human subjects (Fauchier et al., 1998). Although this study does not allow differentiation between effects of onetime and prolonged flecainide application and does not assess AF patients, it still cannot be excluded that the effect of flecainide on beat-to-beat RR variability in our model might, at least in part, be attributable to vegetative mechanisms. Ultimately, it must be speculated that CREM^T/T^ animals very rapidly progress into permanent AF.

## Conclusion

In this study we could show that, by establishing a breeding colony homozygous for the insertion of the CREM-IbΔC-X transgene, one specific phenotype of this animal model, i.e., atrial hypertrophy and spontaneous atrial fibrillation, could be intensified and its development accelerated, with 100% of homozygous mice presenting AF within 10 weeks. At the same time, except for a mild ventricular hypertrophy, no unspecific side effects became obvious, and no increased early mortality up to an age of 6 months appeared. While further studies may help to elucidate effects of the increased gene dose on the arrhythmogenic substrate underlying AF development in the hemizygous model, this study clearly suggests the homozygous model as a feasible and economic animal disease model of AF, with rapid onset of the phenotype and low concomitant mortality.

## Ethics Statement

This study was carried out in accordance with the requirements of relevant German laws and regulations (“Tierschutzgesetz [TSchG]” and “Tierschutz-Versuchstierverordnung [TierSchVersV]”) as well as European Union Directive 2010/63/EU. The protocol was approved by the animal protection commission of the “Landesamt für Natur, Umwelt und Verbraucherschutz (LANUV) Nordrhein-Westfalen.”

## Author Contributions

FM, FS, and JS conceived of the study. KH, BS, BVS, JS, FS, and MS performed the experiments and analyzed and interpreted their data. FS drafted the manuscript, with parts provided by BVS and MS. All authors thoroughly reviewed the manuscript, revised it for important intellectual content, and approved the final version.

## Conflict of Interest Statement

The authors declare that the research was conducted in the absence of any commercial or financial relationships that could be construed as a potential conflict of interest.

## References

[B1] Altarejos J. Y. Montminy M. (2011). CREB and the CRTC co-activators: sensors for hormonal and metabolic signals. *Nat. Rev. Mol. Cell Biol.* 12 141–151. 10.1038/nrm3072 21346730 PMC4324555

[B2] Bukowska A. Felgendreher M. Scholz B. Wolke C. Schulte J. S. Fehrmann E. (2018). CREM-transgene mice: an animal model of atrial fibrillation and thrombogenesis. *Thromb. Res.* 163 172–179. 10.1016/j.thromres.2017.07.033 28807377

[B3] Chelu M. G. Sarma S. Sood S. Wang S. Oort R. J. van Skapura D. G., (2009). Calmodulin kinase II–mediated sarcoplasmic reticulum Ca2+ leak promotes atrial fibrillation in mice. *J. Clin. Invest.* 119 1940–1951. 10.1172/JCI37059 19603549 PMC2701862

[B4] Chugh S. S. Havmoeller R. Narayanan K. Singh D. Rienstra M. Benjamin E. J. (2014). Worldwide epidemiology of atrial fibrillation. *Circulation* 129 837–847. 10.1161/CIRCULATIONAHA.113.005119 24345399 PMC4151302

[B5] Dash S. Chon K. H. Lu S. Raeder E. A. (2009). Automatic real time detection of atrial fibrillation. *Ann. Biomed. Eng.* 37 1701–1709. 10.1007/s10439-009-9740-z 19533358

[B6] Davis J. Maillet M. Miano J. M. Molkentin J. D. (2012). Lost in transgenesis: a user’s guide for genetically manipulating the mouse in cardiac research. *Circ. Res.* 111 761–777. 10.1161/CIRCRESAHA.111.262717 22935533 PMC3466061

[B7] Deshmukh A. Barnard J. Sun H. Newton D. Castel L. Pettersson G. (2015). Left atrial transcriptional changes associated with atrial fibrillation susceptibility and persistence. *Circ. Arrhythm. Electrophysiol.* 8 32–41. 10.1161/CIRCEP.114.001632 25523945 PMC4334691

[B8] Fauchier L. Babuty D. Autret M. L. Poret P. Cosnay P. Fauchier J. P. (1998). Effect of flecainide on heart rate variability in subjects without coronary artery disease or congestive heart failure. *Cardiovasc. Drugs Ther.* 12 483–486. 10.1023/A:1007710301259 9926280

[B9] Fentzke R. C. Korcarz C. E. Lang R. M. Lin H. Leiden J. M. (1998). Dilated cardiomyopathy in transgenic mice expressing a dominant-negative CREB transcription factor in the heart. *J. Clin. Invest.* 101 2415–2426. 10.1172/JCI2950 9616213 PMC508831

[B10] Gallagher A. M. Staa T. P. van Murray-Thomas T. Schoof N. Clemens A. Ackermann D. (2014). Population-based cohort study of warfarin-treated patients with atrial fibrillation: incidence of cardiovascular and bleeding outcomes. *BMJ Open* 4:e003839. 10.1136/bmjopen-2013-003839 24468720 PMC3913087

[B11] James J. Osinska H. Hewett T. E. Kimball T. Klevitsky R. Witt S. (1999). Transgenic over-expression of a motor protein at high levels results in severe cardiac pathology. *Transgenic Res.* 8 9–22. 10.1023/A:1008894507995 10399364

[B12] Kehat I. Hasin T. Aronheim A. (2006a). The role of basic leucine zipper protein-mediated transcription in physiological and pathological myocardial hypertrophy. *Ann. N. Y. Acad. Sci.* 1080 97–109. 10.1196/annals.1380.009 17132778

[B13] Kehat I. Heinrich R. Ben-Izhak O. Miyazaki H. Gutkind J. S. Aronheim A. (2006b). Inhibition of basic leucine zipper transcription is a major mediator of atrial dilatation. *Cardiovasc. Res.* 70 543–554. 10.1016/j.cardiores.2006.02.018 16631626

[B14] Kirchhof P. Benussi S. Kotecha D. Ahlsson A. Atar D. Casadei B. (2016). 2016 ESC Guidelines for the management of atrial fibrillation developed in collaboration with EACTS. *Eur. J. Cardiothorac. Surg.* 37 2893–2962. 10.1093/eurheartj/ehw210 27567408

[B15] Kirchhof P. Marijon E. Fabritz L. Li N. Wang W. Wang T. (2013). Overexpression of cAMP-response element modulator causes abnormal growth and development of the atrial myocardium resulting in a substrate for sustained atrial fibrillation in mice. *Int. J. Cardiol.* 166 366–374. 10.1016/j.ijcard.2011.10.057 22093963 PMC7647839

[B16] Li N. Chiang D. Y. Wang S. Wang Q. Sun L. Voigt N. (2014). Ryanodine receptor–mediated calcium leak drives progressive development of an atrial fibrillation substrate in a transgenic mouse model. *Circulation* 129 1276–1285. 10.1161/CIRCULATIONAHA.113.006611 24398018 PMC4026172

[B17] Müller F. U. Bokník P. Knapp J. Neumann J. Vahlensieck U. Oetjen E. (1998). Identification and expression of a novel isoform of cAMP response element modulator in the human heart. *FASEB J.* 12 1191–1199. 10.1096/fasebj.12.12.1191 9737722

[B18] Müller F. U. Lewin G. Baba H. A. Bokník P. Fabritz L. Kirchhefer U. (2005). Heart-directed expression of a human cardiac isoform of camp-response element modulator in transgenic mice. *J. Biol. Chem.* 280 6906–6914. 10.1074/jbc.M407864200 15569686

[B19] Müller F. U. Neumann J. Schmitz W. (2000). Transcriptional regulation by cAMP in the heart. *Mol. Cell. Biochem.* 212 11–17. 10.1023/A:1007176030884 11108131

[B20] Nishida K. Michael G. Dobrev D. Nattel S. (2010). Animal models for atrial fibrillation: clinical insights and scientific opportunities. *Europace* 12 160–172. 10.1093/europace/eup328 19875395

[B21] Okamoto Y. Chaves A. Chen J. Kelley R. Jones K. Weed H. G. (2001). Transgenic mice with cardiac-specific expression of activating transcription factor 3, a stress-inducible gene, have conduction abnormalities and contractile dysfunction. *Am. J. Pathol.* 159 639–650. 10.1016/S0002-9440(10)61735-X 11485922 PMC1850558

[B22] Riley G. Syeda F. Kirchhof P. Fabritz L. (2012). An introduction to murine models of atrial fibrillation. *Front. Physiol.* 3:296 10.3389/fphys.2012.00296 PMC3429067 22934047

[B23] Rockman H. A. Hamilton R. A. Jones L. R. Milano C. A. Mao L. Lefkowitz R. J. (1996). Enhanced myocardial relaxation in vivo in transgenic mice overexpressing the beta2-adrenergic receptor is associated with reduced phospholamban protein. *J. Clin. Invest.* 97 1618–1623. 10.1172/JCI118587 8601626 PMC507225

[B24] Schulte J. S. Fehrmann E. Tekook M. A. Kranick D. Fels B. Li N. (2016). Cardiac expression of the CREM repressor isoform CREM-IbΔC-X in mice leads to arrhythmogenic alterations in ventricular cardiomyocytes. *Basic Res. Cardiol.* 111:15. 10.1007/s00395-016-0532-y 26818679 PMC4729809

[B25] Seeland U. Selejan S. Engelhardt S. Müller P. Lohse M. J. Böhm M. (2007). Interstitial remodeling in β1-adrenergic receptor transgenic mice. *Basic Res. Cardiol.* 102 183–193. 10.1007/s00395-006-0635-y 17122889 PMC2779411

[B26] Seidl M. D. Stein J. Hamer S. Pluteanu F. Scholz B. Wardelmann E. (2017). Characterization of the genetic program linked to the development of atrial fibrillation in CREM-IbΔC-X mice. *Circ. Arrhythm. Electrophysiol.* 10:e005075. 10.1161/CIRCEP.117.005075 28784605

[B27] van den Berg M. P. Haaksma J. Brouwer J. Tieleman G. Mulder G. Crijns J. G. M. (1997). Heart rate variability in patients with atrial fibrillation is related to vagal tone. *Circulation* 96 1209–1216. 10.1161/01.CIR.96.4.1209 9286951

[B28] Wolf P. A. Mitchell J. B. Baker C. S. Kannel W. B. D’Agostino R. B. (1998). Impact of atrial fibrillation on mortality, stroke, and medical costs. *Arch. Intern. Med.* 158 229–234. 10.1001/archinte.158.3.229 9472202

